# The effect of sildenafil on retinal blood velocity in healthy subjects

**DOI:** 10.1186/s40662-018-0125-y

**Published:** 2018-12-05

**Authors:** Asaf Achiron, Idan Hecht, Chen Juza, Adiel Barak, Zvia Burgansky-Eliash

**Affiliations:** 1 Department of Ophthalmology, Edith Wolfson Medical Center, 62 Halochamim St, 58100 Holon, Israel; 2 Sackler School of Medicine, Tel Aviv University, Tel Aviv, Israel; 3 Department of Urology, Tel Aviv Sourasky Medical Center, Tel Aviv, Israel; 4 Department of Ophthalmology, Tel Aviv Sourasky Medical Center, Tel Aviv, Israel

**Keywords:** Sildenafil, Retinal function imager, Autoregulation

## Abstract

**Purpose:**

It has been suggested that Sildenafil may have beneficial therapeutic effects in the treatment of neurodegenerative disorders. The retinal circulation is of significant interest as a marker of cerebral vascular disease since the retinal and cerebral vasculatures share many morphological and physiological properties, yet only the retinal circulation can be directly visualized. Therefore, our aim was to assess the change induced by Sildenafil on retinal blood velocity.

**Methods:**

Retinal flow velocity was measured 0.5, 3 and 6 h following administration of 100 mg of Sildenafil using the Retinal Function Imager.

**Results:**

No clinical change in either systemic blood pressure or retinal flow velocities were observed. However, when controlling for heart rate and blood pressure, a significant drop in venous flow velocity 6 h following treatment (mean drop 0.3 ± 0.07; 95% CI: 0.44–0.56, *P* = 0.023) was revealed.

**Conclusions:**

In healthy volunteers, retinal venous flow velocity was significantly reduced at the 6-h time point following Sildenafil treatment. No effect was observed on arterial retinal flow velocity.

## Introduction

Sildenafil is a selective inhibitor of the intracellular enzyme phosphodiesterase-5 (PDE5) and is widely used as treatment for erectile dysfunction. Inhibition of cyclic guanosine monophosphate (cGMP)-specific PDE5 results in increased levels of cGMP and nitric oxide. This leads to reduced intracellular calcium levels thereby producing smooth muscle relaxation and an increase in blood flow in erectile tissue [1].

The enzyme PDE5 is present in brain tissue, mostly in the cerebellum and hippocampus [2]. Sildenafil has been shown to affect cerebral function and vasculature [3, 4]. It has been suggested that Sildenafil may have beneficial therapeutic effects in the treatment of stroke, subarachnoid hemorrhage, dementia, and neurodegenerative disorders by enhancing angiogenesis [5]. However, several reports have associated Sildenafil with various neurological adverse outcomes. These include transient global amnesia, ischemic stroke, intracerebral hemorrhage and seizures [6–9], further suggesting some influence on brain vasculature and function.

The retinal circulation is of significant interest as a marker of cerebral vascular disease since the retinal and cerebral vasculatures share many morphological and physiological properties, yet only the retinal circulation can be directly visualized [10]. Several correlates have been described between abnormal retinal vasculature changes and cerebral vascular disease [11, 12]. Several studies have demonstrated an increase in retinal vessel diameter following Sildenafil administration [13–15], yet whether this translates to changes in the blood-flow or blood velocity of the retina is not yet clear [16]. Our goal in this pilot study was to evaluate the change induced by Sildenafil on retinal blood velocity using the Retinal Function Imager (RFI), a new, non-invasive functional imaging system capable of assessing retinal function by measuring the blood velocity [17].

## Methods

### Patients

We included patients between the ages of 18 to 30 years. Patients with major systemic or ophthalmic diseases, media opacity and sexual dysfunction were excluded. Approval was obtained from the local ethics committee of Tel Aviv Sourasky Medical Center and all patients gave their written informed consent.

### Setting

All subjects underwent a standardized ophthalmic examination and each subject filled the international index of erectile function questionnaire to confirm that no erectile dysfunction exists [18]. Brachial blood pressure measurements were performed, and retinal blood velocity measurements were acquired at baseline, 30 min, 3 and 6 h following treatment with 100 mg of Sildenafil. Retinal circulation was evaluated using the RFI device.

### Sample size

The sample size needed for this study was calculated using a univariate approach to repeated measures [19]. Difference in the flow parameters were based on our previous reported measurements in 51 eyes of 31 healthy subjects (mean venous velocity (mm/s) was 3.0 (95% CI: 2.7–3.3) and mean arterial velocity (mm/s) was 4.2 (95% CI: 3.9–4.6)) [20]. We aimed to detect a clinical difference in velocity of at least 10% in each timepoint. To become significant with a power of at least 0.80 and α of 0.05, it would have required a total sample size of at least 6 patients with unstructured correlation or in a LEAR model (correlation monotonically decreases with distance between repeated measurements; base correlation = 0.1, decay rate = 0.05; Grand-Mean hypothesis comparing against a known mean value of 3 (venous velocity) or 4 (arterial velocity)). Calculation of sample size was performed using GLIMMPSE online software [21].

### Retinal function imager

**Fig. 1:**
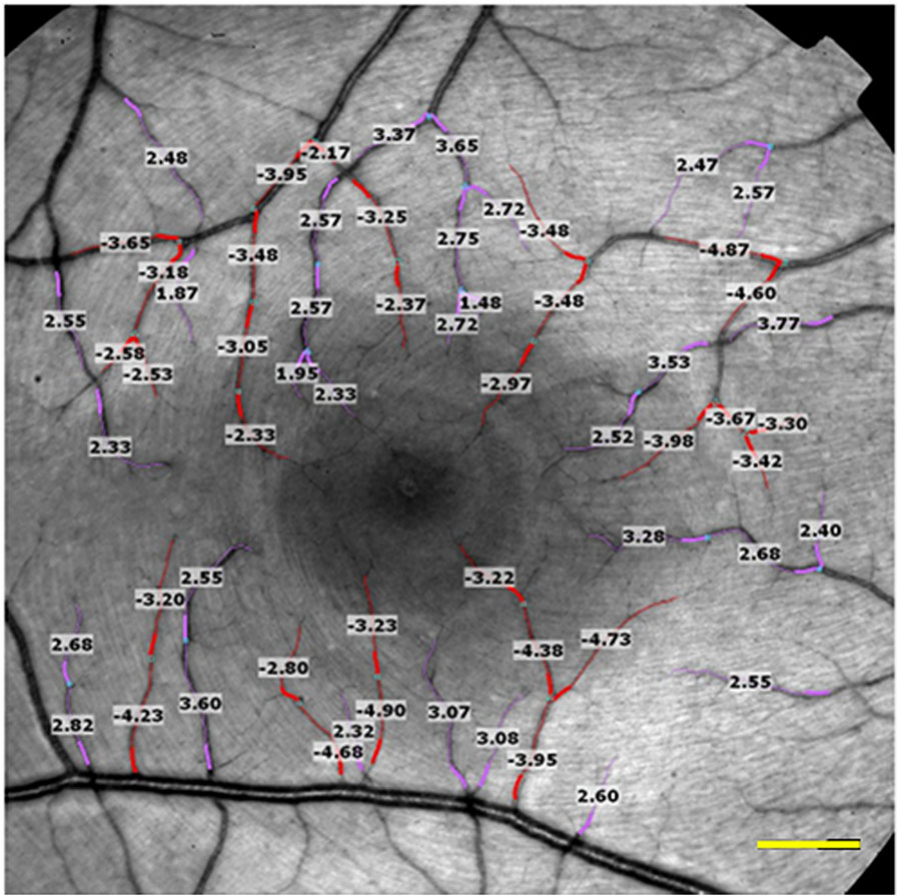
Retinal Function Imager image of the macular region. A representative image acquired during the Retinal Function Imager analysis. Values indicate arterial flow velocity. Red segments are arterioles and their numerical velocity has a minus sign. Purple segments are venules and their numerical velocity is indicated by a plus sign. Scale bar = 500 μm

The RFI is a new, non-invasive functional imaging system capable of measuring retinal blood velocity by direct visualization of the retina. A set of high-resolution images, taken under stroboscopic illumination, were used to detect retinal flow velocity in both venules and arterioles. The system produces 8 flashes of illumination at 17.5 millisecond intervals at a wavelength of 548 nm. It is synchronized to the subject’s pulse phase via a probe attached to the finger or earlobe in order to account for the effect of heart pulsation on flow velocity. Assessment of blood flow is achieved by tracking hemoglobin-filled erythrocytes within blood vessels and conducting in-built software calculations. Analysis of each segment was repeated three times and each segment was assigned a coefficient of variation. Segments with a coefficient of variation exceeding 45% were excluded from the analysis as the segmentation was considered unreliable [22]. Images with over 33% excluded segments were also excluded from the analysis. Previous studies demonstrated high reproducibility of retinal blood flow measurements using the device [23]. A representative image acquired during the RFI analysis is presented in Fig. 1.

### Statistical analyses

Data were analyzed using SPSS software for windows version 22.0 by IBM Inc. All variables were distributed normally, as evaluated by the Shapiro–Wilk test. Changes over time following Sildenafil administration were evaluated using a repeated measure analysis of variance (ANOVA). Bonferroni correction was used for multiple comparisons. Continuous data are presented as mean ± standard deviation. A mixed linear model was used in which the covariance structure of the residuals was modeled. Best covariance structure was found to be of the unstructured type by comparing the − 2 log likelihood of different covariance structures. The flow velocity in either arterioles or venules was taken to be a function of the time elapsed since Sildenafil uptake, with heart rate (HR), and mean arterial pressure (MAP) as covariates. Estimated marginal means of flow velocity were calculated for HR and MAP means (75.95 and 90.77, respectively). The results of the model were as follows: Covariance type = − 2 log likelihood, unstructured = − 19.85, scaled identity = 45.05, diagonal = 44.04 and autoregressive first order = 28.79.

## Results

**Fig. 2:**
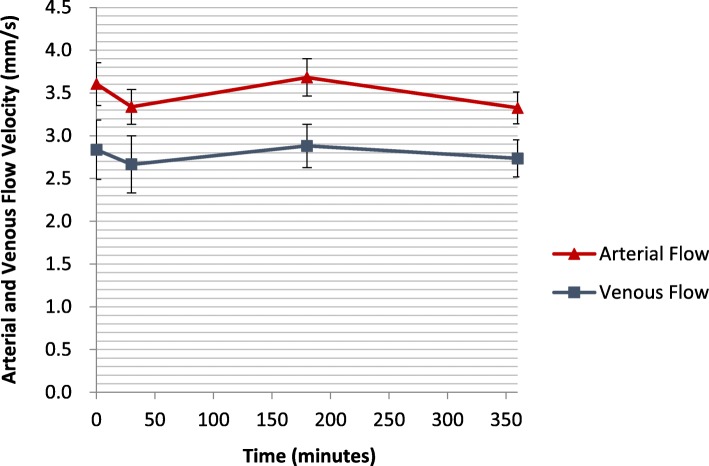
Arterial and venous flow velocities over time. BPM, Beats per minute. mm/s, millimeters per second

This pilot study included 8 healthy male subjects with a mean age of 27.3 ± 1.2 years. The mean venous velocity was 2.86 ± 0.7 mm/s at baseline, 2.66 ± 0.6 mm/s at 30 min, 2.87 ± 0.6 mm/s at 3 h and 2.73 ± 0.5 mm/s at 6 h. The mean arterial velocity was 3.60 ± 1.0 mm/s at baseline, 3.33 ± 0.9 mm/s at 30 min, 3.68 ± 0.7 mm/s at 3 h and 3.32 ± 0.6 mm/s at 6 h. These results are illustrated in Fig. 2. Vein and artery flow velocities correlated well over all time points (*R* = 0.70, *P* < 0.001).Repeated measure analysis of variance revealed no significant difference between vein velocities across all time point (*F* = 0.66, *P* = 0.27). Similarly, no difference was found between arterial velocities across all time points (F = 0.84, *P* = 0.77).When evaluating flow velocity dependence on selected residual covariance structure, the mixed model showed that HR and MAP only affect the venous flow (venous: (HR: F = 1939, *P* < 0.001, MAP: F = 10,753, P < 0.001), arterial: (HR: F = 0.554, P non-significant, MAP: F = 0.672, P non-significant)). Pairwise comparisons of estimated venous flow velocities at sample mean HR and MAP revealed a statistically significant drop in venous flow velocity at 6 h after Sildenafil administration compared to baseline. The mean difference was 0.3 ± 0.07 (95% CI: 0.44–0.56, *P* = 0.023). Similar pairwise comparisons of estimated arterial flow velocities at sample mean HR and MAP failed to reveal a significant change in flow velocity compared with baseline.

## Discussion

The results of this preliminary pilot study revealed that Sildenafil was associated with a significant reduction in venous retinal flow velocity 6 h following treatment when controlling for HR and MAP. However, we did not detect a significant effect on retinal arterial blood velocity or systemic blood pressure in this group of healthy individuals. Venous and arterial flow velocities correlated well over all time points. Sildenafil’s vascular effect has been documented on penile tissue but also on pulmonary, coronary and cerebral vessels [24–26]. Several studies have demonstrated an increase in retinal vessel diameter following Sildenafil administration [13–15], yet whether this translates to changes in the blood flow of the retina is not yet clear [16]. Summary of investigations performed regarding the change in retinal microcirculation induced by Sildenafil is presented in Table 1.

**Table 1:** Summary of studies evaluating the effects of PDE5i on retinal blood flow and vasculature

Author [REF]	Year	Subjects	Method	Conclusion	Overall Demonstrated Trend
Sponsel et al. [34]	2000	12 healthy subjects	Ocular pulse wave tonometer and Doppler flowmetry	Increase in pulsatile ocular blood flow 110 min following administration of 50 mg of Sildenafil	Increased flow
Pache et al. [13]	2002	10 healthy subjects	Retinal vessel analyzer	Increase in retinal arterial and venous diameters 30 min only following administration of 50 mg of Sildenafil, with return to baseline after 120 min	Arterial and venous dilation
Grunwald et al. [35]	2002	15 healthy male subjects	Monochromatic fundus photography	No change in superior and inferior venous diameter or the retinal temporal artery diameter 1 or 5 h following administration of 100 mg of Sildenafil	No effect
Polak et al. [14]	2003	12 healthy male subjects	Retinal vessel analyzer and bidirectional laser Doppler flowmetry	No effect on retinal blood velocity or retinal arterial diameter and an increase in retinal venous diameter and flow following administration of 100 mg of Sildenafil	Venous dilation, arteries and velocity unaffected
Metelitsina et al. [15]	2006	14 male patients with age-related macular degeneration	Monochromatic fundus photographs	Dilatation of major retinal veins 90, 180 and 300 min following administration of 100 mg of Sildenafil	Venous dilation

In our study we used a Retinal Function Imager, a new non-invasive functional imaging system capable of measuring retinal blood velocity with high accuracy [17]. We previously studied the RFI’s reproducibility [27]. The average intra-visit variability as assessed in a subgroup of 20 subjects by means of coefficient of variance was 7.5 ± 3.7%. For measurements from the same subject on different days (inter-visit variability), the average interclass correlation coefficient was *R* = 0.744.

Here, we were also able to assess blood velocity directly. Our results appear to corroborate with previous studies in that a predominantly venous effect exists. We unfortunately did not directly measure the effect of sildenafil on venous diameter but as it has been shown to increase venous diameter by several studies [13–15], it can be safe to assume that this was the case during our trial as well. We did however measure velocity and found it to be steady initially (which combined with larger diameter of vessels might represent higher flow) and a decrease following 6 h. Given constant pressure, as in our trial, when the radius is increased, flow must increase as well in order to maintain equilibrium in the equation. Increased flow through a larger vessel is possible given constant velocity, as Flow = Velocity × A (where A represents the cross-sectional area of the vessel). However, an increase in diameter accompanied by a decrease in velocity, as appears to occur here 6 h following the Sildenafil intake, could result in steady venous flow. These results could represent vasculature reaction to initial increased flow induced by Sildenafil [28]. In addition, we used a mixed linear model in order to isolate Sildenafil’s independent effect on venous and arterial blood flow. The model uses an independent variable (blood flow) and various dependent variables (such as: time since sildenafil administration, HR and BP) as covariates. This method is very useful in order to express the net effect contributed by covariates, while removing possible confounding effects by related variables. When controlling for these factors the results show that venous flow was affected but arterial flow was not. This is not to say that either were associated with HR or BP, merely that only venous flow was affected by Sildenafil after controlling for possible confounding variables.

In rat and mouse models, Sildenafil increases brain levels of cGMP, angiogenesis, and neurogenesis and has been shown to increase cerebral blood-flow in both ischemic and non-ischemic rats [29, 30]. Studies involving humans however, are rare, and their results are less conclusive [31, 3]. Flow in major cerebral blood vessels appears unaffected, yet some perfusion changes were noted [3]. The retinal vasculature is regulated locally by the metabolic needs of the retinal tissue in a similar fashion to cerebral vasculature [28]. Our results suggest that the retinal vessels autoregulatory function was unimpaired by Sildenafil and could further suggest that cerebral vasculature could respond in a similar way.

Regarding limitations, this study was designed as a small pilot study aimed at describing retinal flow changes following Sildenafil administration using novel technology. The small sample size may introduce some bias as the baseline velocity appears to be lower than previously reported [32]. Second, our analysis controlled for heart rate and blood pressure (systolic, diastolic and MAP), however we did not control for intraocular pressure. Although intraocular pressure has been found to affect retinal flow velocity [33], in a previous study performed with the same technology in healthy subjects, no correlation was found between blood velocity and IOP (*n* = 82 eyes) [27]. Previous studies, however, have shown a lack of effect by Sildenafil on intraocular pressure thus we expect any effect to have a limited influence on the results [14]. Third, this study did not include a control group consisting of subjects who were not administered Sildenafil, however, flow measurements were compared, at various times following sildenafil intake, to the flow measurements before ingestion (*t* = 0). Each subject therefore served as their own control by comparing the flow before ingestion (negative control) to post ingestion at various times (test measurements). Fourth, we used the RFI to quantify the retinal venules and arterioles flow by calculating the cross-correlation of moving patterns of erythrocytes over eight consecutive pictures. However, the RFI has not yet been validated against an accepted technique for measuring blood velocity in retinal arteries and veins. Finally, we chose to study the effect of Sildenafil on the retinal vasculature function in healthy subjects in order to avoid other confounders like hypertension, atherosclerosis and medications characteristic for older age, and our results cannot necessarily be generalized to other populations.

## Conclusions

The retinal circulation shares many features with cerebral and other tissues and could provide insights into vascular reactions and disease. Our results corroborate with other studies in that a predominantly venous effect exists on retinal vasculature by Sildenafil and suggest that retinal blood flow-velocity remains constant despite reported vasodilatation, with a late decrease in venous flow-velocity.
